# Immunogenicity of lipid nanoparticles and its impact on the efficacy of mRNA vaccines and therapeutics

**DOI:** 10.1038/s12276-023-01086-x

**Published:** 2023-10-02

**Authors:** Yeji Lee, Michaela Jeong, Jeongeun Park, Hyein Jung, Hyukjin Lee

**Affiliations:** College of Pharmacy, Graduate School of Pharmaceutical Sciences, Ewha Womans University, Seoul, 03760 South Korea

**Keywords:** Gene delivery, Nanoparticles

## Abstract

Several studies have utilized a lipid nanoparticle delivery system to enhance the effectiveness of mRNA therapeutics and vaccines. However, these nanoparticles are recognized as foreign materials by the body and stimulate innate immunity, which in turn impacts adaptive immunity. Therefore, it is crucial to understand the specific type of innate immune response triggered by lipid nanoparticles. This article provides an overview of the immunological response in the body, explores how lipid nanoparticles activate the innate immune system, and examines the adverse effects and immunogenicity-related development pathways associated with these nanoparticles. Finally, we highlight and explore strategies for regulating the immunogenicity of lipid nanoparticles.

## Lipid nanoparticles (LNP): investigating the immune impacts of mRNA/LNP

Drugs consisting of mRNA encapsulated in lipid nanoparticles (LNP), such as the vaccines developed for COVID-19, are poised to have a massive impact in future therapy, but researchers are still learning about their interactions with the immune system. Hyukjin Lee and colleagues at Ewha Womans University, Seoul, South Korea, have reviewed how the formulated RNA/LNP can positively stimulate the immune response to elicit more robust vaccine protection, but also show potential for harm arising from inappropriate or excessive immune activation. The natural response to lipids or foreign RNA may possibly contribute to allergic or even autoimmune conditions. Fortunately, there are strategies for counteracting these adverse effects, including chemical modifications to the RNA, changes in the lipid formulation, or altering the adminiration routes of delivery. More experience with this promising drug class should yield safer and more effective LNP formulations.

## Introduction

Over the past three years, mRNA vaccines have been rapidly developed and utilized worldwide to combat the COVID-19 pandemic. As a result, interest and research on lipid nanoparticles (LNPs), which facilitate RNA delivery into cells, have significantly increased. LNPs are effective RNA carriers that consist of four lipid types, including ionizable lipids, phospholipids, cholesterol, and polyethylene glycol (PEG) lipids^1–3^. The ionizable lipid, which is a crucial component of LNPs, has a tertiary amine structure that enables the encapsulation of RNA and facilitates the transport of RNA to the cytoplasm^4,5^. Phospholipids and cholesterol also play a role in stabilizing the LNPs and aiding endosomal escape, which is critical for ensuring the potency of LNPs^6–8^. PEG lipids enhance the half-life of LNPs, thereby prolonging their circulation time in the body^9–11^.

There are only three RNA/LNP-based drugs approved by the FDA. Following the 2018 approval of amyloidosis siRNA gene therapy, Onpattro, Moderna (Spikevax) and Pfizer’s (Comirnaty) COVID-19 mRNA vaccines were approved in 2021^12–14^. More than two billion people were vaccinated with the COVID-19 vaccine at the end of 2021, generating more than $50 billion in sales. Clinical studies for LNP-formulated gene therapies, including mRNA vaccines, are currently taking place, and as pharmaceutical companies continue to invest in RNA/LNP-based medicines, it is anticipated that more pharmaceuticals will be created and licensed^15^. Since the RNA/LNP platform is still in its early stages, it is unknown how these vaccines impact an individual’s body. After inoculation, several side effects of the COVID-19 vaccine have been reported, and many studies have been conducted on the immune function of LNPs^16^. It appears that the immune function of LNPs exerts contrasting effects. Although it is advantageous when the immune activity of LNPs is positive, there is a possibility that adverse effects could arise as a result of immunological action. Since the immune system is very complex and has various mechanisms, a deeper understanding of the immune system is needed to determine how LNPs affect the body. Moreover, various techniques for controlling LNP effects on the immune system should be established.

In this review, we discuss innate immunity and acquired immunity and their relationship to understand the immune function of LNPs. We also examine the mechanism behind the immune action of lipid nanoparticles. As representative adverse effects of LNP, anaphylaxis, compaction activation-related pseudoallergy reaction (CARPA), and autoimmune disease will also be investigated, along with their mechanisms. Finally, we consider strategies for improving or impairing the immune effects of LNPs. Throughout the course of this review, readers will have the opportunity to thoughtfully evaluate techniques that can control and exploit the immunological activity of lipid nanoparticles.

## Relationship between innate immunity and adaptive immunity

### Innate immunity

The immune system responds quickly to defend the body when it is exposed to antigens. Unlike adaptive immunity, which expresses antigen-specific receptors in T and B lymphocytes, innate immunity is regulated by a limited number of receptors and elicits a protective inflammatory response after antigen exposure. Additionally, innate immunity has a considerable impact on how the adaptive immune response is activated. Innate immunity is mediated by hematopoietic cells such as macrophages, dendritic cells (DCs), neutrophils, eosinophils, natural killer cells (NK), and NK T cells^17^.

A crucial process of innate immunity is the inflammatory response^18^. The inflammatory process is as follows: After a pattern receptor recognizes an antigen on the cell surface, the inflammatory response is first stimulated, an inflammatory factor is released, and inflammatory cells are recruited. The receptor that recognizes the pattern of antigens is called a pattern recognition receptor (PRR), which is present in immune and nonimmune cells, and includes Toll-like receptors (TLRs), C-type lectin receptors (CLRs), retinoic acid-inducible gene (RIG)-I-like receptors (RLRs), and NOD-like receptors (NLRs)^19^. PRRs are activated by inflammatory factors, initiating a signaling cascade and leading to the recruitment of leukocytes^20^.

**Fig. 1:**
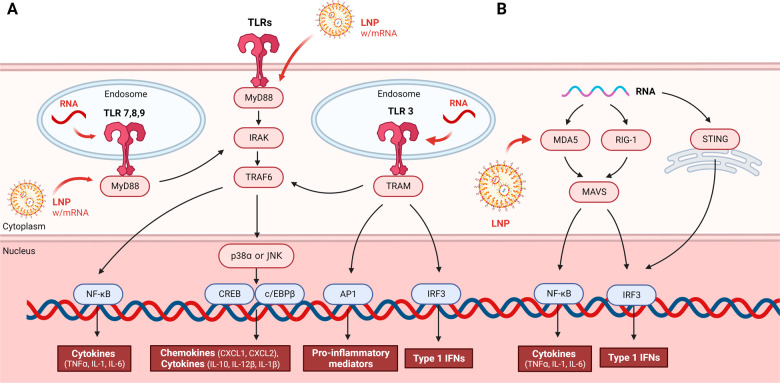
TLR and RLR signaling pathways and the effects of LNPs. **A** TLRs include TLRs 3, 7, 8, and 9 on the endosome surface as well as the rest of the TLRs on the cell membrane surface. When a TLR recognizes a foreign antigen, downstream signals are activated, starting with the MyD88 adapter, except for TLR3. Transcription factors such as AP-1, NF-kB, CREB, c/EBP, and IRF3 ultimately migrate to the nucleus and initiate the innate immune response. When transcription factors are activated, cytokines, chemokines, and type 1 IFNs are activated, and innate immunity occurs. RNA is recognized by endosome TLRs 3, 7, 8, and 9, and LNPs encapsulated with mRNA can be recognized by the MyD88 adapter. **B** When foreign RNA is recognized by MDA5 and RIG-1, RNA helicases in the cytosol and by STING proteins on the surface of the endoplasmic reticulum, the RLR signaling pathway is activated. It activates the transcription factors NK-κB and IRF3 through MAVS, a signaling adapter protein, and promotes cytokines and type 1 IFNs. At this time, LNP can activate MDA5 to trigger immunity.

Of the many PRRs, TLRs are the most frequently studied (Fig. 1A). TLR is a leucine-rich repeat transmembrane protein that recognizes bacterial and viral pathogen-associated molecular patterns (PAMPs). There are various types of TLRs, with TLRs 1, 2, 4, 5, 6, and 11 present in the extralateral environment and TLRs 3, 7, 8, 9, and 10 residing in the endolysosome. When a TLR is activated, transcription factors such as activator protein-1 (AP-1), nuclear factor kappa-light-chain-enhancer of activated B cells (NF-kB), cAMP response element-binding protein (CREB), CCAAT-enhancer-binding proteins (c/EBP), and interferon regulatory Factor 3 (IRF3) migrate to the nucleus and initiate innate immunity. Among them, NK-kB-dependent inflammatory cytokines include TNFα, IL-1, and IL-6^21^. The TLR signaling process is complicated. The transfer of PAMPs and danger-associated molecular patterns (DAMPs) is mediated by myeloid differentiation primary response 88 (MyD88) along with TLR. Except for TLR3, each TLR has a direct or indirect MyD88 adapter. p38α is regulated by TLR2 and TLR4, which activate the transcription factors CREB and c/EBPβ, inducing chemokines (CXCL1 and CXCL2) and cytokines (IL-10, IL-12β, and IL-1β). MyD88-dependent type 1 interferon is expressed by TLR7 and TLR9. TLR7 produces proinflammatory cytokines, and TLR9 promotes the synthesis of IFNα and IFNβ in plasmacytoid dendritic cells (pDCs)^22^.

The RLR family consists of RIG-1, melanoma differentiation-associated protein 5 (MDA5), and the cytosolic RNA helicase, which signals the creation of the proinflammatory cytokine type 1 interferon. RIG-1 and MDA5 have two caspase activation and recruitment domains (Fig. 1B). RIG-1 and MDA5 detect viruses by recognizing RNA. When RIG-1 and MDA5 are stimulated, an RLR binds to the signaling adapter mitochondrial antiviral-signaling protein (MAVS). This binding induces NK-kB and IRF3-mediated type 1 interferon responses. Stimulator of interferon genes (STING), a protein present in the endoplasmic reticulum membrane, can also recognize RNA and induce an interferon response^20,21^.

**Fig. 2:**
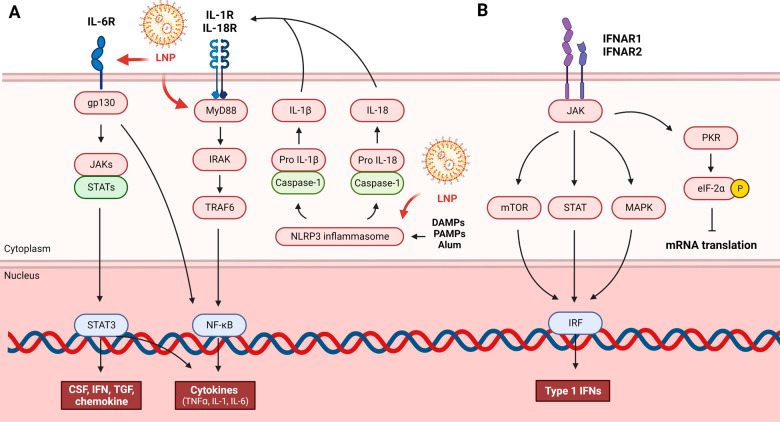
NLR and IFN-mediated signaling pathways and the effects of LNPs. **A** NLRs recognize external substances or stress and induces NLRP3 to generate a nuclear signal complex called the inflammasome to initiate an inflammatory response. Caspase-1 converts pro IL-1 and IL-18 into IL-1 and IL-18, respectively. Caspase-1 products such as IL-1β later promote MyD88-dependent signaling. When IL-6R is activated, STAT3 and NF-κB are activated by downstream signals to secrete cytokines and chemokines. LNP can be recognized by the NLRP3 inflammasome, IL-6 receptor, and MyD88 to initiate immune action. **B** Viral antigens can trigger type 1 interferon. IFNα and IFNβ produced by the innate immune process act on IFNAR1 and IFNAR2 to activate ISG transcription through the ISG promoter. IFNα/β causes cells to enter an antiviral state by expressing ISG. Downstream of IFNAR, various pathways, such as mTOR, JAK-STAT, and MAPK, are activated to express chemokines, cytokines, and antiviral effectors. However, PKR induces phosphorylation of eIF2α, which reduces eIF2 activity and prevents mRNA translation.

NOD-like-receptor (NLR) activates NF-κB and cleaves pro IL-1β and pro IL-18 (Fig. 2A). Whereas TLR is a response to the extracellular environment or endocytic vesicles, NLR is a response to the intracellular environment. NLR recognizes external substances or stress and induces NOD-, LRR- and pyrin domain-containing protein 3 (NLRP3) assembly and activation to generate a nuclear signal complex called the inflammasome and initiate an inflammatory response. Caspase-1 products such as IL-1β later promote MyD88-dependent signaling. Inflammasome activation causes a type of cell death called pyroptosis. NLRP3 is activated by uric acid, asbestos, silica, and alum. Among them, alum is a well-known adjuvant that is related to the NLRP3 inflammasome^17^.

A pleiotropic cytokine, IL-6, plays a role in metabolism, tissue regeneration, and immunity. Rapid IL-6 production contributes to the host’s defense against infection and tissue damage. IL-6 binds to IL-6 receptor (IL-6R) and glycoprotein 130 (gp130), a transmembrane protein that acts as a signal transducer for IL-6, to begin the IL-6 signaling cascade^23^. The IL-6 plasma membrane receptor activates Janus kinase-signal transducer and activator of transcription (JAK-STAT) protein. JAK-STAT signaling can translate extracellular signals into transcription factor signals. Inflammatory genes such as IL-1, TNFα, IL-6, colony-stimulating factor (CSF), IFN, TGF, and chemokines are regulated by a number of transcription factors^22^.

All cells can produce type 1 interferon (IFNα/β), which is generated when a viral substance is recognized by the cell’s PRR^24^. Moreover, type 2 interferon (IFN-γ) is manufactured by immune cells such as T cells, NK cells, and macrophages^25^. IFNα and IFNβ created by the innate immune process act on receptors IFNAR1 and IFNAR2 to activate interferon stimulating gene (ISG) transcription through the ISG promoter (Fig. 2B). IFNα/β causes cells to enter an antiviral state by expressing ISG. Downstream of IFNAR, various pathways, such as mammalian target of rapamycin (mTOR), JAK-STAT, and mitogen-activated protein kinase (MAPK), are activated to express chemokines, cytokines, and antiviral effectors^26^. Type 1 IFNs are versatile antiviral cytokines that influence almost every phase of the immune response to mRNA vaccination, from mRNA expression to DC stimulation to T-cell differentiation. Type 1 IFNs were therefore discovered to be essential regulators of T and B-cell responses evoked by mRNA vaccines^27–29^. Although this activation may be advantageous for mounting an immune response to mRNA vaccines, another immediate effect of type 1 IFNs is the reduction in the translation of eukaryotic initiation Factor 2 (eIF2)α through protein kinase R (PKR) phosphorylation, which reduces eIF2 activity and prevents mRNA translation, inhibiting the synthesis of the immunogen’s protein^30^.

### Relationship between innate immunity and adaptive immunity

The body’s defense mechanism consists of innate and acquired immunity. Acquired immunity is initiated based on the innate immune response^31^. Additionally, regulatory T lymphocytes express TLRs and other innate immune receptors, which blurs the boundaries between innate and adaptive immunity^32^.

TLRs induce innate immunity, which then stimulated the adaptive immune response, with a great effect on the differentiation, memory formation, and antibody formation of CD4 + T cells and CD8 + T cells. TLR activation in APCs, especially in DCs, produces cytokines and upregulates costimulatory molecules essential for inducing T-cell responses. In addition, inflammatory mediators such as TNFα activate DCs, which promote CD4 + T-cell expansion. Moreover, a Th1 response is induced by all TLRs. Some TLRs can also provoke a Th17 response. Th17 cells are proinflammatory T helper cells that secrete IL-17, a potent inflammatory pleiotropic cytokine, and IL-17 causes the production of the proinflammatory cytokines IL-6 and TNF and chemokines KC, MCP-1, and MIP-2^33,34^.

Activating PRRs can upregulate costimulatory molecules or invoke T-cell responses by allowing APCs to secrete cytokines. In addition to indirect methods, T cells directly express PRRs and undergo the innate immune process. MyD88-deficient T cells in TLR-inhibited mice exhibited a reduced TH1 response^35^. IL-6 controls the T-cell response similarly to IL-1, which is known as a T-cell survival activator. Unlike normal conditions, IL-6 induces T-cell expansion under inflammatory conditions^36^ and is involved in T-cell differentiation^37^.

## Immunogenicity of mRNA-LNP complexes

Recent research has shown that RNA stimulates TLRs, specifically TLR3, TLR7, and TLR8, to activate the innate immune system (Fig. 1A)^38,39^. When modified nucleosides, such as pseudouridine (Ψ), 5-methylcytidine (m5C), N6-methyladenosine (m6A), 5-methyluridine (m5U), or 2-thiouridine (s2U), are integrated into the transcript, most TLRs are no longer triggered, and the translation capacity is enhanced^40,41^.

How mRNA and LNPs affect the immune system has been investigated. Among them, one study associated IL-1β, a crucial cytokine in the innate immune response, with the immunological function of mRNA vaccine-encapsulated liposomes (RNA-LPX)^42^. RNA-LPX-induced cytokine secretion was reduced in CD-14-depleted human peripheral blood mononuclear cells (PBMCs), confirming that the RNA-LPX-induced cytokine response was dependent on CD14+ monocytes. IL-1β secretion declined when primary human monocytes were treated with RNA-LPX and the NLRP3 inhibitor MCC950. Hence, the NLRP3 inflammasome and caspase activity are necessary for RNA-LPX-induced IL-1β production (Fig. 2A). IL-1β secretion did not increase when human monocytes were treated with empty LPX but was amplified when treated with R848, a TLR7 and TLR8 agonist. Conversely, treatment with R848 alone did not enhance IL-1β secretion. As a result, both the NLRP3 inflammasome and the TLR7,8 agonist are necessary for IL-1 induction. Essential mediators such IL-1α and IL-1β bind to IL-1R1 and then trigger a signaling cascade that is dependent on MyD88 to generate an inflammatory response (IL-1 receptor type 1). When cytokine secretion was assessed following treatment with anti-IL-1β and RNA-LPX in PBMCs, IL-6, TNF, IL-10, IFN-2, and IL-12p70 were not released. IL-1β stimulates the release of proinflammatory cytokines such as IL-6 and TNF. Moreover, innate immunogenicity varies depending on ionizable lipids. The amount of IL-1β secretion in SM-102 LNPs was significantly higher than that in MC3 LNPs when modRNA-encapsulated SM-102 LNPs and MC3 LNPs were compared^42^.

According to a separate study, the innate immune system uses a different mechanism for LNP rather than the NLRP3 inflammasome. In this investigation, the Pfizer-BioNTech COVID-19 vaccine BNT162b2 (Comirnaty) was employed to confirm its innate immunogenicity^43^. To verify the immunological effectiveness of the vaccine, CD86, an activation factor for immune cells, was evaluated up to seven days after vaccination. CD86 levels were elevated on the first day and dropped to baseline on Day 7 in monocytes, plasmacytoid DCs, and CD103+ migrating DCs. Comparing the activation of immune cells in the draining lymph node (dLN), nondLN, and naïve LN, immune cells were generally activated in the dLN.

IFN-γ is an important cytokine for activating innate immune cells. Serum IFN-γ levels increased six hours after the second dose of the Pfizer vaccine. The importance of IFN-γ was confirmed when interferon stimulating gene (ISG) was diminished when IFN-γ receptors of various immune cells were blocked using antibodies. The innate immune response to the Pfizer vaccination was studied using knockout mice. When *Tlr3*^*-/-*^*, Tlr7*^*-/-*^*, Tlr2*^*-/-*^*, Tlr4*^*-/-*^*, and Tlr5*^*-/-*^ mice were vaccinated with the Pfizer vaccine, no reduction in neutralizing antibody or T-cell response was observed. Treatment of *Asc*^*-/-*^*, Nlrp3*^*-/-*^*, Cgas*^*-/-*^, and *Sting*^*-/-*^ mice with the vaccine also did not affect the number of neutralizing antibodies or the T-cell response. Only Mda^-/-^ mice showed a significant reduction in the number of antigen-specific CD8 + T cells. In addition, compared to wild-type mice, vaccine-treated Mda^-/-^ mice had decreased total blood IFN-α levels, as identified by ELISA. However, the number of neutralizing antibodies was still not reduced. Therefore, MDA5 sensing in response to BNT162b2 vaccination significantly contributes to the induction of a spike-specific CD8 + T-cell response (Fig. 1B)^43^.

To examine whether lipid nanoparticles act as an adjuvant, BALB/c mice were intramuscularly inoculated with influenza hemagglutinin HA (rHA) and coronavirus spike recombinant vaccines together with empty LNP (eLNP) and AddaVax (MF57-like adjuvant), respectively, to confirm neutralizing antibody production. When examined, the eLNP group showed a higher neutralizing antibody titer and T-cell response than the AddaVax group. Therefore, the empty LNP imitates an adjuvant. In addition, eLNPs, except for those constructed with ionizable lipids, did not generate neutralizing antibody titers. When the ionizable lipid was replaced with the cationic lipid 1,2-dioleoyloxy-3-(trimethylammonium) propane (DOTAP), the neutralizing antibody titer was close to baseline. We can conclude that for eLNPs to function as adjuvants, the role of ionizable lipids is critical.

To confirm the innate immune process in which eLNP enhances immunity, MyD88^-/-^ and MAVS^-/-^ mice and wild-type mice were inoculated with rHA-eLNP and HA mRNA-LNP, and the adaptive immune response was confirmed. When MyD88^*-/-*^ mice were inoculated with HA mRNA-LNP, follicular helper T-cell (Tfh) cell proportions and HA-specific GC B-cell numbers decreased (Figs. 1A, and 2A), but rHA-eLNP and MAVS-/- mice displayed similar levels to those of the control mice. In addition, this study revealed that IL-6 is a vital cytokine responsible for Tfh cell differentiation (Fig. 2A). When rHA+eLNP and HA mRNA-LNP were inoculated into mice receiving IL-6-blocking monoclonal antibody, IL-6-deficient mice, Tfh and GC responses were diminished in both groups compared to the control group^44^.

Moreover, another group used RNA-LPX intravenous injections rather than intramuscular injections to study immunity^45^. According to this group, RNA-LPX vaccines cause IFN production that is initiated by TLR7 signaling (Fig. 1A), APC and effector cell activation that is dependent on IFNAR, and a substantial expansion of T cells that are fully functional and antigen-specific. Systemic IFN secretion was dramatically reduced in TLR7^-/-^ mice compared to C57BL/6 wild-type mice, and splenocytes were only weakly stimulated following an intravenous injection of mRNA-LPX. In IFNAR1^-/-^ animals, macrophages did not secrete IFN, whereas pDCs secreted IFN at a moderately reduced rate. By investigating TLR3^-/-^, TLR4^-/-^ and TLR9^-/-^ mice, no reduction in the immune response was observed.

Summarizing the above studies, the notion that lipid nanoparticles act as an adjuvant through when inoculated with mRNA vaccine is supported by: (1) mRNA-LNPs can be detected by TLR, MDA5, and NLRP3; (2) mRNA-LNPs cause the secretion of IL-1β, IFN-γ, and IL-6 through the innate immunity pathway; and (3) mRNA-LNPs promote CD8 + T cell, Tfh, and germinal center (GC) B-cell responses.

## Adverse effects of lipid nanoparticles

**Fig. 3:**
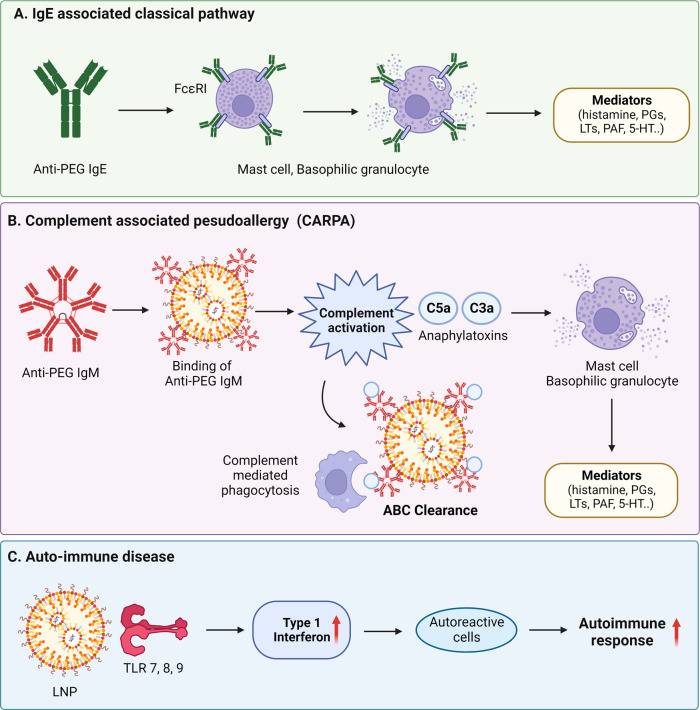
Adverse effects of LNPs. **A** Mechanisms of vaccine adverse responses, including IgE-mediated allergy, IgM-mediated pseudoallergy, and autoimmune reactions. PEGylated LNPs lead the body to generate anti-PEG antibodies, which can have negative side effects. IgE antibodies bind to FcεRI in mast cells or basophilic granulocytes, key cells of the immediate hypersensitivity reaction. Multiple tyrosine kinases are activated as mediators. **B** Upon administration of the PEGylated liposome, anti-PEG IgM in the body binds to the liposome, and this complex causes complement activation via the classical complement pathway and is quickly removed from the blood circulation due to Kupffer cell phagocytosis, which is called the accelerated blood clearance (ABC) phenomenon. This anaphylatoxin induces inflammatory mediators by stimulating macrophages, mast cells and basophils. This mediator binds to receptors of autonomic effector cells, endothelial cells, and smooth muscle cells and induces CARPA through activation. **C** mRNA-LNP-based drugs can cause autoimmunity as follows: (1) mRNA plays the role of autoantigen and triggers the autoimmune process through TLR7; (2) Since LNPs themselves act as an adjuvant, the autoimmune process proceeds through the innate immune response to LNPs; and (3) in the case of mRNA-LNP vaccines, the autoimmune response can be further aggravated because the vaccine itself enhances the immune process.

Side effects related to the immune action of COVID-19 vaccines reported thus far include allergy reactions and autoimmunity. According to the CDC, among the current allergy reactions, fatal anaphylaxis has occurred at a rate of approximately five cases per million vaccine doses administered^16,46^. An increasing number of studies have suggested that adverse reactions to COVID-19 vaccines could include myocarditis^47^, vaccine-induced immune thrombotic thrombocytopenia^48^, IgA vasculitis^49^, autoimmune disorders^50^, and others^51^. It is still unclear whether there is a causative connection between the COVID-19 vaccine and autoimmune symptoms. It is important to comprehend how mRNA-LNP treatment leads to these side effects and how to prevent them. This paper reviews the reported mechanisms of vaccine adverse responses, including IgE-mediated allergy, non-IgE-mediated allergy, and autoimmune reaction (Fig. 3).

## Anaphylaxis

### IgE-associated classical pathway

IgE-mediated allergic reactions appear within 30 min to four hours of receiving the first dose of vaccine, such as urticaria (hives), itching, discomfort, angioedema, breathlessness, burning sensation, and fainting, which can be life-threatening^52–54^. Recent studies point to polyethylene glycol lipid, which is used as an additive in mRNA-LNP vaccines, as the cause of the anaphylactic reaction^55,56^. PEGylation is the introduction of PEG into a drug, which amplifies vaccine stability to enhance plasma half-life and reduces immunogenicity to improve clinical efficacy. PEGylation lowers the clearance rate by the mononuclear phagocytic cells of the liver and spleen and interferes with the binding of the protein opsonin to liposomes^57–60^. Despite these advantages, PEGylated pharmaceuticals lead the body to generate anti-PEG antibodies, which can have negative side effects. Studies on immunological responses to PEGs are necessary because there have been few instances where PEG lipids have been employed in vaccines thus far. PEGylated vaccine allergy occurs in people who have had a previous anaphylactic reaction to PEG. PEG is present in a wide range of everyday items, including toothpaste, cosmetics, and shampoo, so it is possible to produce preexisting PEG antibodies prior to the first vaccine immunization. One study determined that IgG and IgM against PEG were present in 72% of the population^61^. The anaphylaxis reaction is as follows: (1) IgE antibodies bind to FcεRI in mast cells or basophilic granulocytes, key cells of the immediate hypersensitivity reaction; (2) Multiple tyrosine kinases are activated, and the mediators histamines, prostaglandins (PGs), leukotrienes (LTs), tryptase, platelet-activating factor (PAF), heparin, proteases, serotonin (5-HT), and cytokines are secreted.

### Complement-associated pseudoallergy

A typical non-IgE-mediated allergy is C activation-related pseudoallergy (CARPA). Anti-PEG IgM is primarily the cause of CARPA in liposomes^62^. Anti-PEG IgM is produced by the proliferation and differentiation of specific B cells in the marginal zone of the spleen, and this reaction is T-cell independent^60,63,64^. Upon administration of the PEGylated liposome, anti-PEG IgM in the body binds to the liposome, and this complex causes complement activation via the classical complement pathway and is quickly removed from the blood circulation due to Kupffer cell phagocytosis, which is called the accelerated blood clearance (ABC) phenomenon^52,55^. A complement plasma protease that is activated by antibody complexes containing immunoglobulin M (IgM) results in the production of complement protein C5a. Complement products, complement C3a, C4a and C5a, called anaphylatoxins, are the most important mediators of complement activation^55^. These anaphylatoxins induce inflammatory mediators by stimulating macrophages, mast cells and basophils. These mediators bind to receptors of autonomic effector cells, endothelial cells, and smooth muscle cells and induce CARPA through activation^60^. In addition, they are effective regulators of autonomic and cardiovascular organ function in animal studies. Moreover, when they are overexpressed, cardiovascular symptoms and anaphylactic reactions occur. One study also discovered a correlation between pulmonary hypertension and complement activation of PEGylated liposomes^65^.

## Autoimmune diseases

Vaccines and autoimmunity are closely related. Vaccines can trigger an autoimmune reaction in that they can modulate the host-immune response to antigens^66,67^. Various autoantibodies that cause lupus have been observed in vaccinated dogs^68^. Such symptoms are mainly arthritis, vasculitis, central nervous system (encephalitis, demyelination) or peripheral nervous system involvement (Guillain‒Barré syndrome), or thrombocytopenia^67^. Several studies have indicated that the key response of autoimmune diseases is the type 1 interferon response. Increased type 1 IFN levels provoke peripheral tolerance breakdown through the activation of immature myeloid dendritic cells (mDCs), and IFN-matured mDCs activate autoreactive T cells. These cells, together with plasmacytoid DCs, expand autoreactive B cells. IFN-mature DCs also promote apoptosis by activating cytotoxic CD8 + T cells. Capture of apoptotic cells by mDCs and capture of nucleic acid-containing immune complexes by plasmacytoid DCs and B cells enhance the autoimmune response^69^. Furthermore, the process of identifying autoantigens is necessary for the onset of autoimmunity, and endosomal TLR7, TLR8, and TLR9 mostly recognize RNA-related immune complexes. TLR7 and TLR9 recognize viral nucleic acids and nucleic acid-containing immune complexes and stimulate IFN-1 expression to cause systemic lupus erythematosus^69–72^. In addition, the B-cell receptor/TLR7 of autoreactive B cells is activated by RNA and RNA-associated autoantigens, which leads to lupus^73^. In fact, when PBMCs from healthy people were exposed to autoantibodies specific to RNA-binding proteins and anti-double-stranded DNA autoantibodies in the serum of patients with autoimmune disorders, IFN-1 was generated^74^.

### How to control the immunogenicity of LNPs

**Fig. 4:**
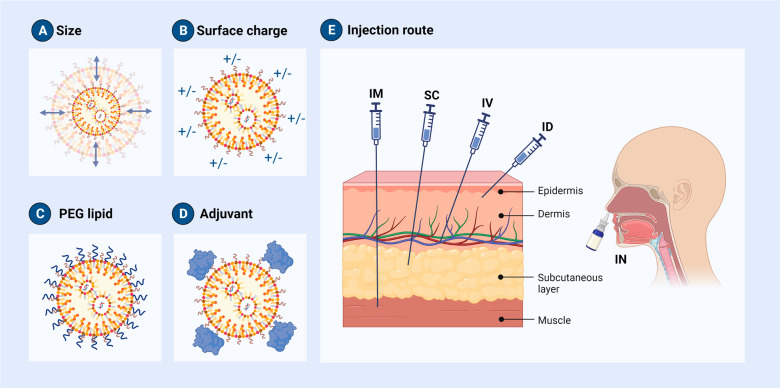
How to modulate the immunogenicity of LNPs. Even a slight modifications can change the properties of an LNP. **A** Altering the molar ratio of PEG or adjusting the formulation rate changes the size of the LNP in the formulation. **B** The charge of the LNP is modified by replacing or adding phospholipids to a charged lipid. **C** Moreover, there are techniques for altering PEG lipids. **D** To improve the efficacy of mRNA-LNP vaccines, adjuvants are being introduced. Adjuvants can be used to enhance the effectiveness of further immunizations. **E** There are several methods for administering mRNA-LNP vaccines, including intravenous (IV), intramuscular (IM), intradermal (ID), subcutaneous (SC), and intranasal (IN). An appropriate route of administration must be determined based on an understanding of the anatomy of the inoculation site and the induced immune action.

As previously mentioned, as the immunological activation in response to mRNA-LNP treatment increases, the body’s defense capability may also rise, but there is a high possibility of the mRNA-LNP complexes causing adverse effects, including allergies and autoimmune diseases. To use mRNA-LNPs as a vaccine, immunity must be boosted, and to minimize adverse reactions produced by the repeated delivery of mRNA-LNPs, immunity must be diminished. Therefore, strategies to modulate the immune system are essential. There are ways to control the immune response to mRNA-LNPs including (1) controlling the composition and characteristics of LNPs; (2) using an adjuvant; and (3) regulating the injection route (Fig. 4).

## Adjusting the composition and characteristics of mRNA-LNPs

The physicochemical characteristics of LNPs can be modified by managing the formulation process. During the LNP formulation process, the four lipids can be altered or added to other components, and the composition ratio of each lipid can be modified. Even a slight difference can change the properties of an LNP. For example, changing the molar ratio of PEG or changing the formulation rate changes the size of the LNP in the formulation^11,75–77^. The charge of the LNP is altered by replacing or adding phospholipids to a charged lipid^78,79^. Moreover, there are techniques for altering PEG lipids^80,81^.

### Modifying the size of LNPs

Hideyoshi Harashima’s research team conducted a study on targeting the lymph node, a key organ that can control the immune response, by adjusting the size of LNPs^82^. The LN is an organ where immune cells assemble, and it can function as a bridge between innate immunity and adaptive immunity^83^. This study team examined whether the LN could be effectively targeted through the subcutaneous injection of LNPs in mice after modulating the LNP size by altering the PEG component ratio of the LNP. The LNP with a low PEG ratio was 200 nm in size, and the LNP with a high PEG ratio was 30 nm in size. As a result, the 30 nm LNP targeted the LN much more accurately than the LNPs of other sizes (100 and 200 nm).

In another study, the size of the LNP was changed by changing the ethanol content, total flow rate during mixing, hold time, and dilution factor in dextrose 5% in water^77^. IgG titers were compared after secondary intramuscular injection into mice and nonhuman primates (NHPs) with LNPs of various sizes encapsulated with mRNA encoding the cytomegalovirus pentamer and glycoprotein gB. In mice, the IgG titer improved as the size approached 100 nm and declined as the size increased. The trend of antibody production capacity according to LNP size, however, was not verified in NHPs.

### Surface charge control

The positive charge of liposomes delivers antigens to APCs and enhances the immune response through DC stimulation^84–86^. For example, N-decyl-N,N-dimethyldecan-1-aminium bromide, a quaternary ammonium lipid, is utilized as an immune adjuvant for mRNA vaccines because it enhances the immune response^87^. However, in both their free and nanoparticle forms, cationic surfactants cause cell damage and the secretion of mediators. It is hypothesized that cationic nanoparticles engage in interactions with cell membranes, resulting in membrane rupture and subsequent Ca2^+^ influx. Degranulation and oxidative stress are brought on by the rise of intracellular Ca2^+^, with cytotoxicity and cell death as a result. Although cationic lipids are effective in promoting cellular uptake and gene delivery, they may also trigger an immune response, leading to inflammation and potential tissue damage^88,89^. As a result, because cationic lipids are toxic, safe ionizable lipids with a neutral charge in the blood were used as gene therapy delivery systems to replace them^90,91^. Studies on the delivery of cationic lipid nanoparticles to the lungs through the addition or substitution of a lipid, such as DOTAP, have been expanding recently^78,79^.

Moreover, Harashima’s research team confirmed the effect of the LNP surface charge on LN targeting, as well as the effect of LNP size. After adding charged lipids to LNPs, negatively charged, neutrally charged, and positively charged particles were subcutaneously administered and the effects were compared. In fact, neutrally charged particles effectively reached the draining LN and the T-cell zone as well^82^.

### Substitution of PEG lipids

Spleen B cells create PEG antibodies when PEG is bound to the surface of LNPs. Anti-PEG IgE and IgM may be responsible for the side effects caused by mRNA-LNP therapeutics. Furthermore, allergic reactions may result from anaphylatoxins produced by the ABC phenomenon that IgM intiates. Regulating the process of the ABC phenomenon is crucial to avoid this process. One strategy is to change the length of the PEG lipid. If the hydrophobic alkyl chain of the lipid is shortened, the PEG lipid can easily separate from the LNP surface^92^. The recognition of PEG attached to liposomes in vivo can lead to the production of antibodies against PEG. One study compared the results of using each LNP on 1,2-dimyristoyl-rac-glycero-3-methoxypolyethylene glycol-2000 (DMG-PEG) and 1,2-distearoyl-rac-glycero-3-methylpolyoxyethylene (DSG-PEG) with 14 and 18 carbon chain lengths, respectively^81^. The ability to produce serum anti-PEG IgM was higher in DSG-PEG LNPs than in DMG-PEG LNPs. The occurrence of complement reactions was also compared in this study. LNPs entered Kupffer cells more quickly when DSG-PEG LNPs were delivered than when DMG-PEG LNPs were delivered, indicating that complement was activated by elevated anti-PEG IgM concentrations.

The molecular weight of PEG is another factor that impacts the production of antibodies against PEG lipids. The PEG content/density on the surface of nanomaterials is one of the most important elements that influences anti-PEG IgM responses because PEG is categorized as an epitope in basic immunology. The half-life increases with PEG molecular weight on the surface of lipid nanoparticles^93,94^. This enhancement of circulation time enriched the concentration of anti-PEG IgM. If the molecular weight of PEG escalates, more antibody against PEG is formed^95^.

To avoid the unforeseen results that PEG can cause, several scientists are exploring PEG substitutes. There are efforts to replace PEG, which is not biodegradable, with biodegradable polymers. In one study, cleavable PEG-cholesterol derivatives were used to reduce the ABC phenomenon caused by PEG^96^. Conventional PEG-1,2-distearoyl-sn-glycero-3-phosphoethanolamine (DSPE) liposomes achieved high ABC and were eliminated by the liver. However, repeated administration of liposomes containing PEG-CHMC, CHEMS, and CHST, cleavable PEG-lipid derivatives, did not result in ABC activity. Moreover, polysarcosine (PSar) is a lipid nanoparticle component that can substitute for PEG lipids. It has fewer systemic interactions and nonspecific interactions and provides longer in vivo circulation times than PEGylated liposomes. Additionally, when comparing Psar with PEG on the same liposome platform, which has equivalent physicochemical features, it prevents the ABC phenomenon. In comparison to PEG-liposomes, PSar-liposomes revealed lower levels of IgM and IgG. In a repeated dose pharmacokinetics study, the PSar coating of liposomes may also help prevent the ABC phenomenon^97,98^.

## Introduction of adjuvants to LNPs

mRNA vaccines have become a promising platform for cancer immunotherapy^99,100^. Applying mRNA-LNP as a cancer vaccination requires a robust immune response^80,101^. To improve the efficacy of mRNA-LNP vaccines, adjuvants are included. Adjuvants boost the effectiveness of further immunizations. To create a local immunocompetent environment at the injection site, adjuvants stimulate innate immune responses. They can change the type of adaptive immune responses that are produced, as well as their strength and effectiveness^102–104^. The following studies applied an adjuvant to LNP.

According to one study, adding additional adjuvants to LNPs improved the immune responses mediated by mRNA^105^. For example, PAM3CSK4 (also known as Pam3), a tri-palmitoyl-S-glyceryl cysteine linked to a penta-peptide, was utilized. Pam3 is a well-known lipopeptide adjuvant that TLR2 and TLR1 can detect. When applied to a mouse tumor model, the mouse survival rate, cellular response, tumor growth inhibition, and humoral response all improved.

Second, by introducing the TLR4 agonist LPS into LNPs, CD8 + T-cell levels and antitumor activity were boosted^1^. Melanoma model mice receiving LPS containing melanoma self-antigen (tyrosinase-related protein 2) mRNA-LNPs lived noticeably longer than negative control mice. As shown by the prolongation of overall survival in a transgenic mouse melanoma model, LPS-containing mRNA-LNPs not only promoted CD8 + T-cell proliferation but also the proliferation of functional killer cells.

A previous study introduced CpG and QS21 adjuvants into LNPs^106^. Adding QS21 and CpG oligodeoxynucleotides (CpG ODN) to LNP had a synergistic effect on both humoral immunity and cell-mediated immunity in the defense of glycoproteins gE of VZV. CpG CpG ODNs, which consist of an essential unmethylated CG dimer found in bacterial and viral DNA, are identified by TLR9 and induce Th1 immune responses. Qs21 is one of the extract components of *Q. saponaria* and is a well-known vaccine adjuvant^107^. Mammalian TLR9 recognizes CpG ODNs, which are composed of a core unmethylated CG dimer present at a high frequency in bacterial and viral DNA and activates Th1 immunological responses. In the liposome-based AS01B adjuvant system, QS21 and monophosphoryl lipid (MPL)-A worked in concert to elicit a high proportion of IgE-specific CD4 + T cells. LNPs enhanced the synergistic adjuvant effect of CpG ODNs and QS21 not only on antigen-specific CD4+ cells but also on CD4+ memory T cells.

In addition to simply adding an adjuvant, researchers have also synthesized an ionizable lipid that acts as an adjuvant^108^. Combinatorial libraries of ionizable lipid-like molecules enable the transport of mRNA in vivo and offer strong and targeted immune activation. By activating protein kinase R, immune responses that activate TLRs and RLRs downregulate the expression of the antigen protein^109^. As a result, these formulations boost antitumor efficacy by limiting systemic cytokine expression and antigen-presenting cell maturation via the intracellular STING pathway rather than through TLRs. LNPs introduced with isocyanide-containing heterocyclic ionizable lipids not only activate DC cells but also exert adaptive immunity and antitumor effects. The most potent candidate formulations lessened tumor growth and lengthened survival in in vivo tumor mouse models of melanoma and human papillomavirus E7.

Other groups have also developed ionizable lipids, which are STING agonist derivatives. Simultaneous innate immune stimulation enhances antigen presentation. A library of nonnucleotide STING agonist-derived amino lipids (SALs) was generated and formed into LNPs for mRNA delivery. SAL12 lipid nanoparticles (SAL12-LNPs) were the most effective at delivering mRNAs encoding the SARS-CoV-2 Spike glycoprotein (S) while activating the STING pathway in DCs. In mice, intramuscular immunization with SAL12 S-LNPs against SARS-CoV-2 generated stronger neutralizing antibodies than Pfizer’s COVID-19 vaccine LNPs (ALC-0315)^110^.

Another study developed an immunostimulatory ionizable lipid structure by adding imidazole (DOG-IM4)^111^. DOG-IM4 comprises an imidazole-based ionizable head group, a dioleoyl lipid tail, and a small, flexible polyoxyethylene spacer between the head and tail. When stored as liquid in phosphate buffered saline at 4 °C, DOG-IM4 LNPs can deliver influenza HA mRNA in mice and macaques, provoking a strong immune response and providing remarkable durability to the encapsulated mRNA. It was hypothesized that certain characteristics of the lipid’s imidazole head group are responsible for the improved immunization.

## Consideration of injection route or target

There are several methods for administering mRNA-LNP vaccines, including intravenous (IV), intramuscular (IM), intradermal (ID), subcutaneous (SC), and intranasal (IN)^112–117^.

### Intravenous injection

The IV route of drug administration is injecting a drug with a needle directly into a vein. The drug passes directly into the systemic circulation without the delay associated with absorption processes, delivering its therapeutic effect faster than any other route, making it the best approach to provide a dose quickly and precisely^78,79,118^. In a research investigation on an mRNA-LNP-based anticancer vaccine, powerful, long-lasting, and systemic CD8 T-cell responses were needed to effectively attack tumors. Improved T-cell responses and anticancer immunity were induced by the IV delivery of mRNA vaccines^114^, which is due to its ability to mobilize the substantial APC pools that are present in the spleen^80^.

### Intramuscular injection

Intramuscular injection is the most commonly used administration method to induce immunity when administering a vaccine^119^. Pfizer, Moderna and COVID-19 vaccines used this method of administration^120^. IM injections are administered into the denser, muscular fascia that lies under the subcutaneous tissues^121^. However, there is a delay before the therapeutic effect starts when a medicine is injected intramuscularly, as the drug must be absorbed before it can enter the bloodstream^112^.

### Subcutaneous injection

Subcutaneous administration, also known as hypodermic administration, involves injecting pharmaceuticals under the skin into the adipose layer beneath the dermis. It is typically performed on the outside of the arm, thigh, or abdomen, and it allows for lesser injection quantities than the IM route^122^. The SC route often results in slower absorption kinetics than the IM route because the SC tissue is less irrigated than the muscle^112^. The lymphatic targeting of liposomes has been studied primarily using SC administration. Liposomes administered subcutaneously cannot enter the bloodstream directly. Instead, they are either absorbed by the lymphatic capillaries that drain the injection site or they remain there. Normally, the first 12 h after an injection are when lymphatic absorption takes place^123^.

### Intradermal injection

As there are numerous antigen-presenting cells in the skin, this area stimulates strong immunological reactions. The epidermis contains APCs such as Langerhans cells (LCs), which facilitate the capture of antigens, whereas the dermis has more dendritic cells^124–126^. In clinical trials, SC and IM immunizations produce fairly equal immune responses, whereas ID immunization produces larger immunological responses than IM injection^116,127,128^. The proper positioning of the needle is a substantial obstacle to ID delivery. Moreover, SC/ID injections may cause more pain than IM injections^129^. Due to the possibility of local irritation, induration, skin discoloration, inflammation, and granuloma formation following SC and ID delivery, the CDC advises that inactivated vaccines containing an adjuvant be injected into a muscle^119,130^.

### Intranasal injection

Vaccines can also be administered via intranasal (IN) immunization, which is a painless, noninvasive method. M cells, which transport particulate antigens to the nasal lymphoid tissue through transcytosis, mediate the uptake of vaccinations given orally. Dendritic cells are highly abundant in the nasal cavity and can drive potent local and systemic immune responses against infections^131^. Although the effectiveness of the vaccine through IN injection is not optimal, the antigen invades through the mucosa and is effective in forming specific immunity such as IgA. By releasing IgA into the nasal cavity and intestinal tract, nasal vaccination produces both systemic and mucosal immunity in the respiratory and genital systems, with fewer side effects than other inoculation methods^115,132^.

### Comparison of mRNA-LNP inoculation routes

Since the environment of each inoculation site is different, to control the immune action of mRNA-LNPs, an appropriate route of administration must be determined based on an understanding of the anatomy of the inoculation site and the induced immune action. Several studies have compared different administration methods based on the lipid nanoparticle delivery mechanism. Kranz et al. described that IV mRNA-lipoplex (LMP) vaccination represents a superior route of administration compared to the SC or ID routes, inducing high-level T-cell responses that demonstrated profound antitumor efficacy in syngeneic tumor models^45^. Broos et al. also indicated that the IV vaccination of mice with LMP based on RNAiMAX (cationic lipid) resulted in the generation of potent T-cell responses. LMP IV administration, but not SC or IM adminstration, provokes robust antigen-specific T-cell responses. The activation of ovalbumin (OVA)-specific T lymphocytes was assessed following the IV, SC, and IM administration of LMPs carrying 5 g OVA mRNA. The highest increase in OVA-specific CD8 + T lymphocytes was observed after IV administration of OVA mRNA, including LMPs^133^. Another study disclosed that the conflicting effects of type 1 IFN signaling on the strength of vaccine-evoked T-cell responses rely on mRNA-lipoplex administration and are controlled at the T-cell level. The researchers used the same mRNA and lipoplexes (DOTAP/DOPE and RNAiMAX) to confirm the opposite effects of type 1 IFN on T-cell immunity during IV versus SC delivery. IFNAR^-/-^ mice were IV and SC injected with the mRNA-lipoplex vaccine. After inoculation, IV-injected mice exhibited moderately diminished T-cell IFNAR signaling compared to that of control mice, and SC-injected mice showed little immune response in control mice but was induced in IFNAR^-/-^ mice. Therefore, IFNAR signaling in T cells promotes T-cell immunity when administered intravenously but inhibits it when administered subcutaneously^117^.

Anderluzzi et al. examined the immunogenicity of a self-amplifying mRNA encoding the rabies virus glycoprotein enclosed in various nanoparticle platforms (solid lipid nanoparticles [SLNs], polymeric nanoparticles [PNPs], and LNPs) to examine the effect of the administration route on RNA vaccine potency. Three different delivery methods were utilized, including intramuscular, intradermal, and intranasal delivery. Ionizable lipid nanoparticles showed higher immunogenicity than other delivery platforms in terms of anti-rabis virus glycoprotein (RVG) IgG titers, RVG-specific CD8 + T cell numbers, and RVG-specific CD4 + T cell numbers. Generally, IM injection was on an equal level with or greater than ID injection. IN injection, however, demonstrated minimal vaccination effectiveness^115^.

In addition to simply comparing the routes of administration, there were cases in which different routes of administration produced synergistic effects. Mao and colleagues created a vaccination approach known as “prime (IM) and spike (IN)”, which makes use of preexisting immunity brought on by primary IM vaccination (prime) to trigger mucosal immunological memory in the respiratory system using unadjuvanted intranasal spike boosters (spike). They demonstrate that, in comparison to only IM and IN injection groups, the prime and spike group exhibited robust resident memory B and T-cell responses and improved systemic immunity, and the technique protected mice with incomplete immunization against lethal SARS-CoV-2 infection^134^.

## Conclusion

In vivo genomic medicine has made considerable progress in the last decade, especially in the field of RNA-LNP-based therapeutics. The success of mRNA-LNP COVID-19 vaccines has demonstrated the potential of the LNP platform, generating substantial revenue and providing hope for future treatments. The LNP industry based on gene therapy and editing has high potential for development, and ongoing clinical trials suggest that DNA and mRNA vaccines will continue to dominate treatment paradigms until 2036^15^. However, with these advancements, it is crucial to comprehend and regulate the immunological effect of RNA-LNPs. This paper provides an answer to this question and encourages further research in the field, providing insights for safer and more effective treatments in the future.

The recognition of PRRs (such as TLRs, CLRs, RLRs, and NLRs) can trigger immune responses, including type 1 interferon, which can affect adaptive immunity. We introduced how innate immunity can interact with mRNA-LNP and influence adaptive immune responses. Based on previous research, we collected publications that examined the immunological responses elicited by mRNA-LNPs. mRNA-LNP is detected by TLR, MDA5, and NLRP3 and simulates IL-1β, IFN-γ, and IL-6 production via the innate immunity pathway. Additionally, it promotes CD8 + T cell, Tfh cell, and GC B-cell responses. We also explored the potential adverse effects of mRNA-LNP through immunological mechanisms, such as PEG-lipid-induced IgE-mediated anaphylaxis and IgM-mediated CARPA. mRNA-LNP is also recognized as a self-antigen and triggers autoimmune diseases. Finally, we emphasized that modifying the size of mRNA, LNP charge, incorporating adjuvants, or altering the route of LNP administration could modulate the immune response.

Investigations on the biological mechanisms of innate immunity have made considerable progress from the late 19th to the early 20th century to the current level of knowledge. Charles Janeway’s discovery of the innate immune receptor and the Nobel Prize awarded to Jules Hoffmann, Bruce Beutler, and Ralph Steinman for their contributions to the understanding and research of the immune system created a foundation for ongoing research^135–137^. Although there are still unknown innate immune mechanisms and relationships being investigated^138–141^, understanding the immunological effects of LNPs represent a considerable breakthrough that has the potential to offer individualized treatment and immunizations^142^. Further research is necessary to uncover additional underlying mechanisms and develop LNPs to generate the desired immunological responses. Continuous efforts in this area can contribute to a better understanding of the immune system and provide innovative therapeutic options for various diseases.
